# PARTICIPANT VIEWS OF THE TASKS AND CHARACTERISTICS OF SUPPORTIVE SUPPORT BROKERS IN SELF-DIRECTION PROGRAMS

**DOI:** 10.1093/geroni/igz038.860

**Published:** 2019-11-08

**Authors:** Ellen K Mahoney, Grace Oh, Carmen Morano, Kevin J Mahoney, Andrew DeVellis

**Affiliations:** 1 Connell School of Nursing, Boston College, Chestnut Hill, Massachusetts, United States; 2 School of Social Welfare, University at Albany, State University of New York, Albany, New York, United States; 3 Boston College School of Social Work National Resource Center (NRCPDS), Lauderdale-by-the-Sea, Florida, United States; 4 Simmons School of Social Work, Boston, Massachusetts, United States

## Abstract

This qualitative study draws on 76 ethnographic case studies with Cash and Counseling participants, examines what participants and their caregivers saw the support broker doing, and looks at what the participants found helpful and less than helpful. Participants and their caregivers saw support broker duties as Coaching, Problem Solving, Advocacy and Monitoring. Equally important was how the support broker performed these roles. Six attributes that mattered to participants were: Familiarity, Supportive Relationship, Proactive Engagement, Responsiveness, Knowldge and Cultural Friendliness. These findings from the participant and caregiver perspective have great import for the training of present and future care managers and support broker, and have implications for regulatory and even licensure requirements. These results can be a first step in constructing a quality framework for self-directed supports and services.

